# Satellite stories: capturing professional experiences of academic health sciences librarians working in delocalized health sciences programs

**DOI:** 10.5195/jmla.2018.214

**Published:** 2018-01-02

**Authors:** Jackie Phinney, Amanda Rose Horsman

## Abstract

**Objective:**

Health sciences training programs have progressively expanded onto satellite campuses, allowing students the opportunity to learn in communities away from an academic institution’s main campus. This expansion has encouraged a new role for librarians to assume, in that a subset of health sciences librarians identify as “satellite librarians” who are permanently located at a distance from the main campus. Due to the unique nature of this role and lack of existing data on the topic, the authors investigated the experiences and perceptions of this unique group of information professionals.

**Methods:**

An electronic survey was distributed to health sciences librarians via two prominent North American email discussion lists. Questions addressed the librarians’ demographics, feelings of social inclusion, technological support, autonomy, professional support, and more.

**Results:**

Eighteen surveys were analyzed. While several respondents stated that they had positive working relationships with colleagues, many cited issues with technology, scheduling, and lack of consideration as barriers to feeling socially included at both the parent and local campuses. Social inclusion, policy creation, and collection management issues were subject to their unique situations and their colleagues’ perceptions of their roles as satellite librarians.

**Conclusions:**

The results from this survey suggest that the role of the academic health sciences librarian at the satellite campus needs to be clearly communicated and defined. This, in turn, will enhance the experience for the librarian and provide better service to the client.

## INTRODUCTION

Academic health sciences librarians at satellite campuses are a unique category of information professionals. All health sciences librarians encounter challenges that are not experienced by other academic librarians, such as keeping apprised of new developments in specialized point-of-care products that require regular instructional updates, focusing on evidence-based practice that requires in-depth and up-to-date searching capabilities, needing to constantly evaluate the collection for its accuracy and relevancy, and responding to rapid turnaround times for information to support health care decisions. Adding to these demands, however, satellite librarians are often geographically separated from the day-to-day workings of their colleagues and their main libraries in a delocalized academic environment.

In such settings, the curriculum is typically managed by the parent institution, whereas the program itself takes place at a satellite campus hosted at another university or college entirely (hereafter referred to as the host institution). In these circumstances, the librarian may encounter hurdles when supporting the curriculum of the parent institution within the framework of the host institution. They are also at risk of feeling disconnected from coworkers, and their job descriptions may vary depending on institutional arrangements. For these and other reasons, it is important to understand more about their day-to-day work situations.

The authors also sought to understand this topic for personal reasons. Supporting health sciences curricula at our respective satellite campuses has afforded us wonderful opportunities, but it has also presented challenges. In looking to the literature, we did not encounter survey data on librarians in our exact circumstances. Instead, the literature primarily described library services for satellite campuses with a focus on program success or student experience, solo librarians working in hospitals, and satellite librarianship in unspecified subject areas.

Beginning with the work of our Canadian colleagues, we found that Fyfe et al. discussed their provision of library services to distributed medical students in British Columbia, Canada. In their program, librarians are dispersed throughout local hubs in their respective regions and provide continuous service in those locations. They noted that the success of their program largely depended on centralized planning and governance, local autonomy, trust, respect, and careful communication [1]. Nicholson and Eva described student satisfaction with their satellite library program at the University of Lethbridge in Alberta, Canada [2], and Ismail contributed to this area of knowledge through her investigation of the library instruction preferences of satellite campus students during their orientation activities and studies in the United States [3]. At our respective satellite campuses, we like to monitor our patrons’ satisfaction to ensure that we are reaching them effectively.

Our positions afford us the opportunity to collaborate with colleagues from different areas of expertise, which is also demonstrated by the hospital librarianship literature. Miles discussed her experience of acting as a solo librarian in a hospital library and described the various organizational projects and teams she participated in as part of her role. She noted that she supported one residency program along with community members and employees, and although her patron population is different than that of our targeted participants, her experience of multitasking and striving to create interorganizational networks likely rings true for most librarians who are delocalized [4]. These networks can be helpful in the event of work absence or the inability to address a patron’s question, and Resnick described the complication when solo hospital librarians require vacation time in her editorial piece [5].

Delving into the literature on satellite librarianship in general academia, we found that Bottorff et al. explored issues surrounding the collaboration, communication, and networking among solo librarians in a multicampus academic library systems that were not specific to the health sciences. Their data revealed that isolation and communication barriers were common issues confronting satellite academic librarians [6].

Judging by the literature, we found that survey data pertaining to health sciences librarians who support university or college programs at satellite campuses were lacking. Important themes are discussed throughout the literature, but we could still stand to gain a greater sense of these librarians’ roles and how they perceive their situations. For instance, what challenges do they face? Whom do they collaborate with, and are they included in shared decision making? Do they feel socially included and supported? By giving this population the chance to provide anonymous answers to such questions, we gain an honest sense of who they are as professionals as well as their successes and pitfalls. Therefore, the purpose of this study was to solicit the experiences, barriers, and facilitators, as well as information about the levels of social inclusion of librarians who support delocalized health sciences programs at satellite campuses.

## METHODS

To achieve the purpose of this study, we utilized a survey instrument. For this study, our population of interest was academic health sciences librarians at satellite campuses. As we also work as satellite librarians for academic health sciences programs, we capitalized on our own experiences to develop the survey and examine the data. However, in an effort to reduce bias, we did not fill out the survey ourselves, even though we met the inclusion criteria. Care was also taken to consider all angles of academic satellite librarianship, and external reviewers analyzed the survey tool. In the survey, open-ended questions were included to extract the respondents’ voices without influence from the investigators, and respondents were encouraged to explain themselves in free-form text boxes.

We carefully crafted survey wording, and to ensure clarity for the respondents, the “parent institution” was defined throughout as the academic institution that oversees the program’s curriculum, while the “host institution” was defined throughout as the place where the satellite campus is located in a different geographical location than the parent institution. The survey was divided into several sections to address participant demographics, their administrative environment, and the workplace implications for both the satellite library and the parent institution. The questions revolved around the themes of social inclusion, administrative inclusion, and services. To view the full survey, please see the supplemental appendix.

A convenience sampling method was used to recruit volunteer participants who would be readily available to participate in the study. After being piloted among colleagues and finalized, the survey was distributed electronically using Opinio survey software on the Medical Library Association and Canadian Health Libraries Association/Association des bibliothèques de la santé du Canada email discussion lists.

Participant suitability was determined by answering “yes” to three introductory survey questions: (1) are you currently working as a librarian for an academic institution (i.e., university or college)?; (2) do you provide library support for a health sciences curriculum?; and (3) do you work at a satellite campus of the program you support (i.e., is your primary workplace in a different geographic location than the parent institution that oversees the program’s curriculum)? Participants were given the option to respond to the survey in English or French, and once the survey was closed, the data from Opinio was exported into Microsoft Excel and SPSS software for analysis. Research ethics boards at both Dalhousie University and the Université de Moncton approved this study.

## RESULTS

In total, 18 completed surveys were analyzed, with an additional 4 that were incomplete or ineligible due to inclusion criteria. While the option to respond in either English or French was given, only 1 completed survey was submitted in French, while the other 17 completed surveys were in English. The majority of respondents were from the United States (10 out of 18), 1 was from Lebanon, and the remaining 7 were from Canada. The parent institution managed most of the libraries and librarians in this survey (12 out of 18), particularly in the United States, whereas the others were managed by the host institution or, in one case, by a hospital. Most respondents were not unionized (12 out of 18).

Looking at inclusivity, results showed that most respondents had positive comments regarding how they felt included by the host institutions (10 out of 18). While a few respondents said that they had very little interaction with host institution employees, others offered enthusiastic confirmation of being treated well, noted that “special effort” had been made by the host institutions, and stated that they had even been “adopted” by the local library as their own.

The majority of the librarians’ workspaces (15 out of 18) were in the library rather than in another location such as a hospital or in the faculties they supported. Those who worked in a library said they were more likely to be able to rely on other library staff for assistance, which was particularly true at sites where the parent institution oversaw the library. That said, most respondents (11 out of 18) said there was no one to replace them if they were absent from work.

The majority of respondents (11 out of 18) noted that they participated in on-site collaborations with local employees. Regardless of employer (host or parent institutions), librarians were involved in meetings at the host institutions (12 out of 18). Unfortunately, the exact nature of these meetings was not elaborated upon in the survey data.

When asked about creating and implementing library policies, the respondents expounded on many stories and frustrations. The underlying theme was that the specific situations that satellite librarians encountered often fell outside any existing policies at the parent or host institution. One respondent said, “I have created my own policies because the main campus library policies do not address many of my needs.” Another respondent noted that “each location has its own culture.” Some respondents described taking the autonomous distinction and simply “manag[ing] the library with minimal contact with the parent institution” or stating that they just informed the necessary chains of command of the policies that they have created for their situations.

When considering inclusion by the parent institution, results showed that respondents were often involved in collaborative efforts and meetings with the parent institutions. One respondent said, “I am considered part of the team and included on pretty well all decision making meetings (sometimes too many given my responsibility to the host institution),” while another said, “Yes, but due to staffing issues it can be difficult to attend events.”

Meetings and inclusion were largely facilitated by communication technology infrastructures, and having local technical support aided in this as well. However, distance was mentioned many times by respondents in their elaborated responses as being the greatest barrier, and an expectation that they would commute to the parent institution to participate in events was mentioned. This distance barrier was also prevalent in comments made about collaborating with others at the parent institutions. Responses contained a mixture of sentiments ranging from “our parent institution is very open to collaboration across all of the satellite campuses”; to “difficulty in arranging meetings”; “geographically complicated, quite different patron profiles”; and “they ask for our input and feedback and then generally ignore it.”

When the technological infrastructure was not sufficient to replace travelling, the level of inclusion became less positive. Those librarians who had access to supportive technology found participating in meetings was more difficult than being able to attend in person, as they ended up feeling “forgotten about” or that the meetings were irrelevant to the satellite setting. When asked about travel to the parent institution, several respondents said that it was encouraged, although time and money were common barriers. Travel, time, scheduling, and money all came up again when respondents were asked if they felt socially included by the parent institutions. One respondent said that she was invited to events but felt perceived as an outsider and, therefore, tended to “feel a bit alienated at such events.” Another felt the same awkwardness in not having a lot in common with the staff they worked with on a regular basis.

Scheduling was a common problem as well, with comments mentioning that “timing is often inconvenient”; there were “schedule conflicts”; and events were “usually not on days I am on campus.” On a positive note, half of those who left comments felt included as members of the teams, noting that “they make every effort to make us feel as if we are part of a larger organization” and they were “included on pretty well all decision making meetings.”

The librarians noted that they provided services to patrons of the host institutions (16 out of 18), whereas very few provided distance services to patrons located at the parent institutions. Looking at collection development, satellite librarians indicated that, at the very least, they made recommendations for book purchases, while some indicated that they were masters of their own on-site collections. In addressing electronic book acquisitions in particular, 8 out of 14 respondents who answered this question stated that electronic book purchases were out of their hands, with the parent institution handling most of the purchasing.

When asked about technical challenges, 10 out of 18 respondents provided comments about technical challenges that they encountered while being apart from the parent institution. Due to the nature of dealing with separate institutions, some respondents said that it can be confusing when trying to obtain information technology (IT) support. One noteworthy comment was: “I have to become very technically knowledgeable because otherwise the local IT and the university IT tend to bounce me back and forth between them. Now that I’ve created a rapport with both, they tend to do it less.” Another echoed this independence with: “the challenges and advantages are the reason for my position: namely that as a satellite library we are left to our own devices to navigate technical issues.”

Others continued to express the technical difficulties caused by being separate from the parent institution, especially when they did not have administrative access on their computers to manage the necessary resources. One respondent elaborated in more detail that “communicat[ing]/meeting with main campus via technology, lack of general IT support, [and] different expectations of undergraduate vs health sciences users” were all problematic.

When addressing professional support, 16 of the 18 respondents elaborated on their experiences. Email discussion lists were a popular form of professional support, as they were mentioned by 13 of the 16 respondents who answered this question. Twelve out of the 16 respondents stated that they relied on colleagues, which included fellow satellite librarians and librarians from the parent institution, host institutions, conferences, and hospital libraries. Webinars (10 out of 16 responses received) were the third-most mentioned form of support, and 1 respondent elaborated on this further with: “webinars—because I mostly work alone, I use webinars heavily to keep my skills up to date.” Lastly, conferences and professional associations were listed by a few respondents.

## DISCUSSION

The existing literature presented in the introduction gave successful program indicators and provided some reflection on personal experiences of solo librarians. This study delved deeper to look for larger patterns. From this study, we have learned that Fyfe et al.’s [1] themes of centralized planning and governance, local autonomy, trust, respect, and careful communication along with Bottorff et al.’s [6] themes of collaboration, communication, and networking were experienced by satellite academic health sciences librarians. Our study revealed similar themes in the attitudes and perceptions of this population.

The participants in this study indicated a lack of cohesion and policy planning due to differing local situations as well as challenges with communication due to time, money, and technology. Local autonomy seemed to be an ideal situation for many, especially for collection planning, where participants mostly noted a lack of control in final decision making. This lack of autonomy can cause hardships for satellite librarians who are trying to do their jobs in a timely fashion and cannot always wait for decisions to come from parent institutions. These holdups can have implications for service delivery, which can trickle down to client satisfaction and possibly affect accreditation outcomes for health sciences curricula.

Many participants showed their willingness to participate at both the host and parent institutions. Barriers were encountered at both levels, often due to not feeling part of either institution. The qualitative responses regarding social inclusion seemed to take on more frustrated tones, which was surprising given the more positive quantitative responses. There were indications of either institution sending out invitations to social activities, but how respondents were treated at the events and whether logistical considerations were made in the invitations hampered the social inclusion attempts. When the respondents gave positive quantitative responses to their levels of social inclusion, they equally gave positive responses to collaboration, communication, and networking.

When respondents spoke positively about interpersonal collaboration, the same respondents were more likely to report feeling included in social activities. That said, inclusion in meetings was more important to the respondents than inclusion in social activities. Distance was a major factor, as it often created logistical issues such as time, money, and appropriate scheduling of the event. As found in Bottorff et al.’s study [6], satellite librarians were more likely to visit the parent institution than be visited by their colleagues from the parent institution. As echoed in this study, the burden often fell on the satellite librarians’ shoulders to participate without always having the proper technological or financial support. Not being able to fully participate with one’s colleagues can leave a librarian feeling out of the loop, especially when one is already missing out on the daily goings-on of one’s colleagues at the parent institution’s library. Fostering respect through actions by making arrangements that respect the satellite librarian’s time and situation would go a long way toward inclusion of the satellite librarian.

The greatest facilitator to social inclusion appeared to be the special efforts made by the institutions to include the librarians. Special efforts included having the roles and expectations of the satellite librarian clarified and understood by the host and parent institutions, especially at the library level, so that meetings and collaborations were more inclusive. A clear understanding of all parties’ responsibilities can impact how colleagues interact with the satellite librarians, which, in turn, leads to a richer experience for the professionals as well as for their clients.

Networking is an important aspect of satellite librarians’ work. This is evident when talking about the professional support that the librarians sought out. Given the feeling of being alone in their work or even misunderstood by colleagues at the local and parent institutions, they reached out through other avenues, such as other satellite librarians, conference colleagues, and webinars. That said, there was also a distinct emphasis on local, in-person support. While networking is a popular tool for librarians regardless of their stations, it is especially crucial for those who are in delocalized environments and require it to thrive.

We acknowledge some limitations of this study. Given that convenience sampling was used, our results might not be generalizable to all satellite librarians supporting academic health sciences curricula. We recognize that location, budgetary variations, and differences in curricula might have impacted respondents’ perspectives.

Given the unknown exact number of delocalized academic health sciences librarians who participate in the two email discussion lists used for recruitment, we consider eighteen completed surveys to be a reasonable sample size. That said, the respondents indicated that email discussion lists are a common professional-support tool, so distributing this survey through email discussion lists was indeed one of the best ways to recruit their responses. Future studies on this subject could employ additional recruitment techniques, such as targeting special interest groups or making use of convenience sampling at a conference.

Future studies could also include questions related to reporting structure, monitoring of library statistics, and workload. Further development of survey questions or even in-person interviews would allow a greater understanding of the realities that academic health sciences librarians who work at satellite campuses face. The participants in this study were mostly limited to selecting multiple choice answers, while having the opportunity to elaborate where possible. Many librarians did indeed elaborate to give further meaning to and understanding of their experiences, and it would be very interesting to see the richness of answers if participants could discuss their experiences using interview or focus group methodologies.

In our study, the respondents were not asked about their perspectives of success as librarians in satellite health sciences campuses but rather about their feelings and experiences in their positions. It would be interesting in the future to discover how success is measured by these librarians. If we were to base success by the program descriptions from the literature, it would be fair to say that many of respondents’ programs were successful, while many still required work. This study will hopefully spark discussion in academic health sciences libraries with satellite counterparts to ensure that all librarians feel that they have the tools and support to succeed.

The goal of exploring the professional situations of academic health sciences librarians at satellite campuses was well met thanks to the candidness of the participants. These librarians face many challenges as well as many opportunities for growth. We hope that the results of the survey will aid in giving a voice to these professionals to know that they are not alone and experience common challenges. Seeing much congruency in the responses gives hope that solutions can be found. Such recommendations could include providing more local autonomy and decision-making power; having better technical systems in place; being respectful of the time, obligations, and geographic challenges in organizing social activities and work-related meetings; and tapping into external (and internal) social networks to provide a greater sense of comradery. This study enables a better understanding of the experiences, barriers, and facilitators as well as the levels of social inclusion of librarians who support delocalized health sciences programs at satellite campuses.

## Supplemental File

Appendix Survey
