# Correction

**DOI:** 10.5195/jmla.2018.459

**Published:** 2018-04-01

**VOLUME 106**

**106(1) January, page 80**

Phinney J, Horsman AR. Satellite stories: capturing professional experiences of academic health sciences librarians working in delocalized health sciences programs. J Med Libr Assoc. 2018 Jan;106(1):74–80. DOI: http://dx.doi.org/10.5195/jmla.2018.214.

The author photos in the hypertext markup language (HTML) version are reversed. The author block should be:

**AUTHORS’ AFFILIATIONS**


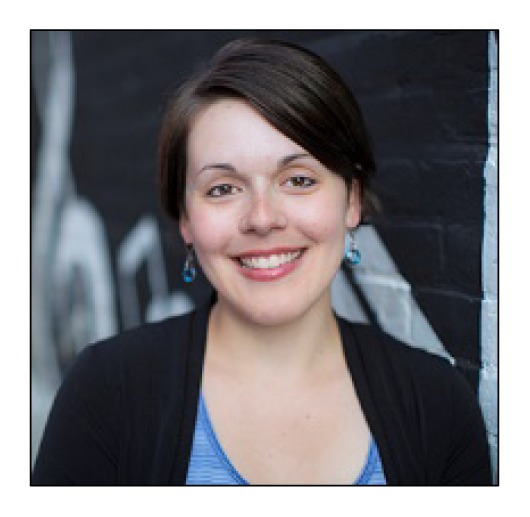


**Jackie Phinney,** j.phinney@dal.ca, Instruction/Liaison Librarian, Dalhousie Medicine New Brunswick, Dalhousie University, Saint John, NB, Canada


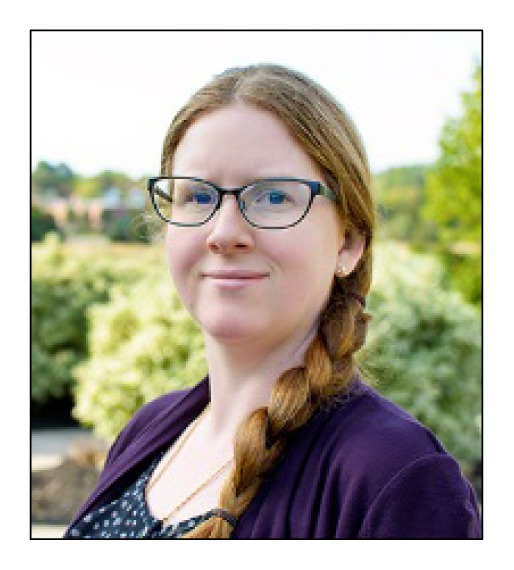


**Amanda Rose Horsman, BA (hons), MLIS, AHIP, PhD Student**, amanda.horsman@umoncton.ca, orcid.org/0000-0002-8281-2752, Bibliothécaire de médecine universitaire/Academic Medical Librarian, Centre de formation médicale du Nouveau-Brunswick, Moncton, NB, Canada

