# Supplementary material for: Satellite stories: capturing professional experiences of academic health sciences librarians working in delocalized health sciences programs
Source: J Med Libr Assoc. 2018 Jan 2;106(1):74–80. doi: 10.5195/jmla.2018.214 (PMC5764596; doi:10.5195/jmla.2018.214)
Supplement: Appendix [file jmla-106-74-s001.pdf]

JMLA Journal of the Medical Library Association

SC

Supplemental content to
J Med Libr Assoc. Jan;106(1):dx.doi.org/10.5195/jmla.2018.214
www.jmla.mlanet.org
© Phinney, Horsman 2018

Satellite stories: capturing professional experiences of academic health sciences librarians working in delocalized health sciences programs

Jackie Phinney; Amanda Rose Horsman, BA (hons), MLIS, AHIP

APPENDIX

Survey

Recruitment email

Apologies for cross-posting

Hello,

You are being invited to participate in a survey of academic health sciences librarians working at satellite campuses of universities or colleges. The purpose of this survey is to gather information regarding your experiences in your role as a satellite health sciences librarian at a distributed campus. The findings will contribute to original research in this area and inform the professional practice of health sciences librarians working with, and at, satellite campuses. For the purposes of this study, "academic health sciences librarians" refers to those professionals with a master of library and information studies degree (or equivalent) who are currently working for a university or college and providing library support for a curriculum in the health sciences (medicine, nursing, pharmacy, allied health, etc.). The term "satellite campus" refers to academic campuses that are in a different geographic location than the main institution (aka, "parent institution") that oversees the health sciences curriculum that you support.

You are being asked to fill out an online questionnaire, which will take approximately 15–20 minutes to complete. The survey is completely voluntary, and you may choose to stop participating at any time. There are no direct benefits from participating in this survey, but your contributions will help shape what is known about satellite librarianship in support of academic health sciences programs.

Data from incomplete surveys will not be included in the final analysis, and the survey is anonymous and no identifying information will be collected. The information will be stored in a password-protected account, and only the researchers will have access to this information. The survey will remain open until December 19, 2016. This study has been reviewed for its adherence to ethical guidelines and approved by the Research Ethics Board at Dalhousie University [File # xxxxx].

The link to the survey is found here: [INSERT LINK]

If you have any questions about the survey please feel free to contact:

Jackie Phinney
Librarian, Dalhousie Medicine New Brunswick
Dalhousie University
Email: j.phinney@dal.ca

Amanda Horsman
Bibliothécaire, Centre de formation médicale du Nouveau-Brunswick
Université de Moncton
Email: amanda.horsman@umoncton.ca

Thank you in advance for taking the time to reply. We look forward to hearing from you! If you would like information about publication of results, feel free to contact us.

JMLA Journal of the Medical Library Association

SC

Supplemental content to
J Med Libr Assoc. Jan;106(1):dx.doi.org/10.5195/jmla.2018.214
www.jmla.mlanet.org
© Phinney, Horsman 2018

Informed consent form

Study Name: Exploring the Professional Landscape of Academic Health Sciences Librarians at Satellite Campuses

Researchers: Jackie Phinney, librarian, Dalhousie Medicine New Brunswick of Dalhousie University and Amanda Horsman, bibliothécaire, Centre de formation médicale du Nouveau-Brunswick of Université de Moncton

Purpose of the Research: The purpose of this survey is to study academic health sciences librarians working at satellite campuses (university or college) and to gather information regarding your experiences in your role as a satellite health sciences librarian. The findings will contribute to original research in this area and inform the professional practice of health sciences librarians working with, and at, satellite campuses.

What You Will Be Asked to Do: You are being asked to fill out a questionnaire, which will take approximately 15–20 minutes to complete.

Risks and Discomforts: No risks or discomfort from your participation in the research are foreseen.

Voluntary Participation/Withdrawal from the Study: Your participation in the study is completely voluntary, and you may choose to stop participating at any time. Responses are anonymous; therefore, once the final responses have been submitted, they cannot be removed from the data set. Data from incomplete surveys will not be included in the final analysis. Your decision not to volunteer will not influence your relationship with the researchers, Dalhousie University, or the Université de Moncton.

Confidentiality: The survey is anonymous, and no identifying information will be collected. Survey data will be collected through the Dalhousie University secure server using Opinio survey software. Researchers will keep all your survey responses confidential, survey completion is anonymous, and there will be no link to your IP address or any personal identifiers. Individual quotations may be used in the presentation of results; however, quotations will not be linked to individuals.

Questions about the Research? If you have questions about the research in general or about your role in the study, please feel free to contact Jackie Phinney by email (j.phinney@dal.ca) or Amanda Horsman by email (amanda.horsman@umoncton.ca). The plan for this study has been reviewed for its adherence to ethical guidelines and approved by the Research Ethics Board at Dalhousie University [File # xxxxx].

We thank you for your time!

By clicking on the "Next" button below, I agree to participate in this survey. I am aware that I am free to withdraw from the survey at any time up until I submit my responses to the survey.

[Next Button]

Survey instrument

Section 1: Introductory questions

1. Are you currently working as a librarian for an academic institution (i.e., university or college)?
   a. Yes
   b. No

Supplemental content to
J Med Libr Assoc. Jan;106(1):dx.doi.org/10.5195/jmla.2018.214
www.jmla.mlanet.org
© Phinney, Horsman 2018

2. Do you provide library support for a health sciences curriculum (medicine, nursing, nutrition, kinesiology, pharmacy, etc.)?
   a. Yes
   b. No

3. Do you work at a satellite campus of the program(s) you support (i.e., is your primary workplace in a different geographic location than the parent institution that oversees the program's curriculum)?
   a. Yes
   b. No

Answer YES to all, proceed to Section 2. If NO to any, survey ends.

Section 2

1. Where are you located?
   a. Canada
   b. United States
   c. Other (please elaborate)
   d. Prefer not to answer

2. Which of the following is your employer?
   a. Parent institution of the program(s) you support
   b. Host institution where the satellite campus is located
   c. Both (please elaborate)

Section 3

1. Do your patrons have access to an on-site library?
   a. Yes
   b. No

If YES, proceed to Section 4. If NO, proceed to Section 5

Section 4

1. Which institution oversees the on-site library?
   a. Parent institution
   b. Host institution
   c. Other (please elaborate)

Supplemental content to
J Med Libr Assoc. Jan;106(1):dx.doi.org/10.5195/jmla.2018.214
www.jmla.mlanet.org
© Phinney, Horsman 2018

2. Where is your primary workspace located?
    a. Library
    b. Other (please elaborate)

3. Do you rely on staff at the on-site library to provide circulation or reference services to your patrons?
    a. Yes
    b. No

4. Do you have any opportunities to collaborate with or assist other librarians at the on-site library?
    a. Yes
    b. No

If YES:

4.1. Have you encountered any barriers while doing this? Please explain.

5. Are you invited to participate in managerial or administrative meetings at the on-site library?
    a. Yes
    b. No

6. What are your experiences with establishing/negotiating library policies and implementing changes at the on-site library? Please elaborate.

Section 5

1. Do you provide library services to patrons of the host institution (i.e., patrons who are enrolled in academic programs that you do not officially support)?
    a. Yes
    b. No

2. Are you invited to participate in managerial or administrative meetings at the host institution?
    a. Yes
    b. No

3. Do you feel socially included in events at the host institution? Please elaborate.

Journal of the Medical Library Association

Supplemental content to
J Med Libr Assoc. Jan;106(1):dx.doi.org/10.5195/jmla.2018.214
www.jmla.mlanet.org
© Phinney, Horsman 2018

Section 6

1. Do you have any opportunities to collaborate with or assist other librarians at the parent institution?
   a. Yes
   b. No

If YES:

1.1. Have you encountered any barriers while doing this? Please elaborate.

2. Are you invited to participate in managerial or administrative meetings at the parent institution, either remotely or in person?
   a. Yes
   b. No

3. Do you use video/teleconferencing technology to communicate with colleagues at the parent institution?
   a. Yes
   b. No

If YES:

3.1. Do you feel engaged/included during these meetings? Please elaborate.

4. Do you feel socially included in events at the parent institution? Please elaborate.

5. Do colleagues from the parent institution visit you at the satellite campus?
   a. Yes
   b. No

6. Are you encouraged to travel to the parent institution? Please elaborate.

7. Do you provide library services at a distance to patrons who are located at the parent institution?
   a. Yes
   b. No

8. Are you included in discussions held by the parent institution to decide which databases to purchase/cancel in your subject area(s)?
   a. Yes
   b. No

Supplemental content to
J Med Libr Assoc. Jan;106(1):dx.doi.org/10.5195/jmla.2018.214
www.jmla.mlanet.org
© Phinney, Horsman 2018

9. Do you make purchases for an on-site print collection? Please elaborate.

10. Do you purchase electronic books? Please elaborate.

11. If you are absent from work, is there another librarian to fill in for you?
    a. Yes
    b. No

12. If technical support is needed, is there someone local who can assist you?
    a. Yes
    b. No

13. What technical challenges or advantages have you encountered in your role as a satellite librarian? Please elaborate.

14. Please list the top 3 ways you receive professional support in your role (i.e. colleagues, professional email discussion lists, webinars, etc.).

15. Please share other duties on which you wish to elaborate.

Thank you for your time.
